# *Astragalus membranaceus* Extract Attenuates Inflammatory Cytokines and Matrix-Degrading Enzymes in Human Chondrocytes: A Novel Nutraceutical Strategy for Joint Health

**DOI:** 10.3390/cimb47090731

**Published:** 2025-09-09

**Authors:** Alessia Mariano, Rosario Russo, Anna Scotto d’Abusco, Fabiana Superti

**Affiliations:** 1 Department of Biochemical Sciences, Sapienza University of Rome, 00185 Rome, RM, Italy; alessia.mariano@uniroma1.it; 2 Giellepi S.p.A., Via G. Verdi, 41/Q, 20831 Seregno, MB, Italy; rosario.russo@giellepi.it; 3 Association for Research on Integrative Oncology Therapies, (ARTOI) Foundation, Via Ludovico Micara, 73, 00165 Rome, RM, Italy

**Keywords:** *Astragalus membranaceus* root extract, inflammation, chondrocytes, joint diseases, matrix metalloproteases, nutraceuticals

## Abstract

The dried root extract of *Astragalus membranaceus*, also known as *Astragali radix*, is widely used in traditional Chinese medicine for its multiple health benefits and well-established safety profile. *Astragalus* root extract exhibits several bioactive properties, including anti-inflammatory, antioxidant, antiviral and hepatoprotective effects. Due to its unique features, it is being investigated in a novel application as a complementary remedy in the management of joint disorders. In this study, we evaluated the effect of *Astragalus membranaceus* hydroalcoholic root extract (0.01 and 0.1 mg/mL) in vitro on the HTB-94 cell line, a well-known model for studying inflammatory pathways in human chondrocytes. The mRNA modulation levels were measured by quantitative real-time polymerase chain reaction (qRT-PCR), while the protein secretion levels were assessed using an Enzyme-Linked Immunosorbent Assay (ELISA). Results obtained demonstrated that this extract is able to decrease the tumor necrosis factor-α (TNF-α)-induced inflammatory response by downregulating both the mRNA expression and release of the pro-inflammatory mediators Interleukin-6 (IL-6), Interleukin-1β (IL-1β) and Interelukin-8 (IL-8), as well as matrix metalloproteases, including Matrix Metalloprotease-3 (MMP-3), Matrix Metalloprotease-13 (MMP-13) and A disintegrin, and metalloproteinase with thrombospondin motifs 5 (ADAMTS-5). Moreover, the interleukin and matrix metalloprotease production was also assessed in non-TNF-α-stimulated cells, revealing that the extract did not alter the basal levels of these mediators. Finally, our findings highlight the potential benefits of *Astragalus membranaceus* extract, both in terms of its favorable safety profile and its efficacy mitigating joint inflammatory responses. These results support the potential of this extract as a nutraceutical agent for joint health support.

## 1. Introduction

Joints are anatomical structures that connect bones within the skeleton, providing both stability and mobility. These structures are frequently affected by painful and sometimes disabling conditions, largely driven by inflammatory processes that result in the degradation of articular cartilage [1]. Inflammation is a hallmark of various joint disorders, which are broadly classified under the term arthritis. The causes of joint inflammation are multifactorial [2]. It may be due to autoimmune mechanisms, as observed in rheumatoid arthritis (RA), juvenile idiopathic arthritis, neuropathic arthropathy and seronegative spondyloarthropathies, or it may be associated with aging, as in the case of osteoarthritis (OA) [3,4]. Among these conditions, OA is the most common joint disorder, affecting several diarthrodial joints, such as the hands, knees and hips [5]. Due to the growing proportion of elderly individuals, its medical relevance is increasing, particularly in Western countries. OA affects 7.6% of the global population and is now considered a disease of the entire joint structure, including the articular cartilage, subchondral bone, ligaments, capsule and synovial membrane [6]. OA is typically classified into two forms: primary OA, which is idiopathic and associated with aging, obesity, genetic predisposition and low-grade systemic inflammation; and secondary OA, which results from trauma, surgical interventions and congenital joint abnormalities [5,7]. If left untreated, inflammatory arthropathies result in progressive joint damage and deformity [8,9], leading to a chronic and irreversible degenerative process. Since inflammation is the mechanism driving both the onset and progression of degenerative joint diseases, early and appropriate anti-inflammatory intervention is essential to prevent structural damage, disease advancement and disability. The structure of cartilage tissue, which is mainly constituted by glycosaminoglycans, proteoglycans and collagens, depends on a homeostasis due to the balance between anabolic and catabolic processes. Pro-inflammatory stimulation can alter this balance, causing the hyperproduction of joint-degrading enzymes, matrix metalloproteases (MMPs) and articular damages [9,10]. For this reason, the current pharmacological treatments are mainly based on the administration of steroidal or nonsteroidal anti-inflammatory drugs (NSAIDs) [11]. However, the long-term administration of these agents is associated with significant side effects, including gastrointestinal [12], renal [13], cardiovascular [14] and hepatic [15] toxicity. In addition, NSAIDs may also cause allergic reactions [16] and increase the risk of hypertension and fluid retention [17]. Given these limitations, there is growing interest in identifying novel approaches capable not only of controlling symptoms but also of modifying the progression of chronic joint diseases. In this context, nutraceuticals, particularly those derived from botanicals and plant extracts, have emerged as promising sources of bioactive compounds with anti-inflammatory properties [18,19].

Among these, *Astragalus*, a genus belonging to the *Fabaceae* (or *Leguminosae)* family, is widely distributed throughout the temperate regions of the world from Central and Southwestern Asia to South America and Africa, and it is known for its broad spectrum of biological effects [20,21,22]. The dried root of *Astragalus membranaceus* (Fisch.) Bunge var. *Mongolicus* (Bunge) Hisao, or *Astragalus membranaceus* (Fisch.) Bunge, commonly referred to as *Astragalus radix*, is a key component in traditional Chinese medicine and is widely used in functional food, health products, cosmetics and veterinary applications [22,23,24]. Several studies have demonstrated that *A. membranaceus* extract possesses well-known anti-inflammatory [25], immunomodulatory [26,27] and antioxidant [22,28] properties, therefore making it particularly suitable for the treatment of those pathologies with inflammatory etiologies. In addition, emerging studies have demonstrated its antiviral, anti-aging, anti-tumor, hepatoprotective, nephroprotective and cardiovascular protective properties [29,30]. *A. membranaceus* extract has also shown anti-inflammatory efficacy in intestinal and allergic pathologies [25,31,32,33]. A summary of clinical trials reporting activities for A. membranaceus can be found in the review by Durazzo et al. [34]. *A. membranaceus* extract has also been shown to reduce the adverse side effects of specific therapies used to treat diseases, particularly those related to food intake problems [35]. Furthermore, according to the Chinese Food and Drug Administration (FDA), *A. membranaceus* extract can also be utilized as a functional food [36]. Given its favorable safety profile, further investigation into the potential anti-inflammatory action of *A. membranaceus* extract in joint-related diseases is warranted.

In this study, we employed the in vitro HTB-94 cell line, an established experimental model for studying inflammatory pathways in human chondrocytes, to evaluate whether *A. membranaceus* extract can inhibit the TNF-α-induced expression of pro-inflammatory interleukins and downregulate MMP production. Previously, three different *A. membranaceus* extracts were analyzed and compared to natural *A. membranaceus* extract, finding that one of the three, the commercial sample named Axtragyl^®^, showed a higher content of bioactive compounds [37], prompting us to use this extract. Moreover, Durazzo et al. found that several compounds present in *A. membranaceus* extract have anti-inflammatory properties [34]. The aim of this study was to explore the potential of commercial *A. membranaceus* extract (Axtragyl^®^) as a safe and effective nutraceutical candidate for modulating joint inflammation and preventing cartilage degradation.

## 2. Materials and Methods

### 2.1. Cells

The human chondrosarcoma cell line HTB-94 (SW1353; American Type Culture Collection) was cultured at 37 °C in Dulbecco’s modified Eagle’s medium (DMEM) (HyClone, Logan, UT, USA) supplemented with 10% heat-inactivated fetal bovine serum (FBS) (Invitrogen, Paisley, UK), 2 mM glutamine, nonessential amino acids, 100 IU/mL penicillin and 100 μg/mL streptomycin.

### 2.2. Materials

The hydroalcoholic extract derived from the dried roots of *Astragalus membranaceus* (marketed as Axtragyl^®^) was generously supplied by Giellepi (Milan, Italy). Recombinant tumor necrosis factor-α (TNF-α) was obtained from PeproTech EC Ltd. (29 Margravine Road, London, UK). The ELISA Pro kits for human IL-6, IL-1β and IL-8 (CXCL8) were purchased from Mabtech AB (SE-131 28, Nacka Strand, Sweden). The SensiFAST™ cDNA Synthesis Kit and SensiFAST™ SYBR Hi-ROX Kit were acquired from Bioline Meridian Biosciences (5171 Wilfong Road, Memphis, TN, USA). ELISA kits for human ADAMTS5, MMP-13 and MMP-3 were sourced from Fine Biotech Co., Ltd. (Wuhan, China).

### 2.3. Assessment of Cell Viability (MTS Assay)

To assess any potential cytotoxic effects of *A. membranaceus* extract, the viability of HTB-94 cells exposed to this compound was analyzed using an MTS 3-[4,5-dimethylthiazol-2-yl]-5-[3-carboxymethoxyphenyl]-2-[4-sulfophenyl]-2H-tetrazolium)-based colorimetric assay (Promega Corporation, Madison, WI, USA) in accordance with the manufacturer’s protocols.

In brief, 1.5 × 10^4^ cells per well were plated in a 96-well plate with 100 μL of medium. Cells were either left untreated (control, CTL) or exposed to *A. membranaceus* extract at concentrations of 0.01, 0.1, 0.5 and 1 mg/mL for 24, 48 and 72 h. After each incubation period, a 100 μL of MTS reagent was added to each well. Absorbance was measured at 492 nm after a 3 h incubation using a microplate reader (NB-12-0035, NeBiotech, Holden, MA, USA). Cell viability was expressed as a percentage of dead cells using the following formula: % dead cells = 100 × [(OD treated/OD control) × 100].

### 2.4. Cell Treatment

HTB-94 cells were plated at the required density and allowed to adhere. After adhesion, the cells were serum-starved (DMEM containing 1% FBS) before stimulation and/or other treatments.

After 24 h of serum starvation, cells were untreated (CTL) or treated with *A. membranaceus* extract (0.01 and 0.1 mg/mL). After 1 h of incubation at 37 °C, cells were left untreated (CTL) or exposed to TNF-α 10 (ng/mL) in the presence or absence of the *A. membranaceus* extract (0.01 and 0.1 mg/mL) for the required time.

In order to analyze the pro-inflammatory cytokine or MMP mRNA expression levels, the cells were harvested after 30 min of stimulation with TNF-α, and then the mRNAs were processed for qRT-PCR.

To quantify the pro-inflammatory cytokine and MMP protein secretion, cell supernatants were collected and analyzed by ELISA, after 1 h or 24 h of TNF-α stimulation, respectively.

### 2.5. RNA Extraction and Reverse Transcription

Total RNA was isolated from both untreated and *A. membranaceus* extract-treated HTB-94 cells using the Blood/Tissues Total RNA extraction kit (Fisher Molecular Biology, Trevose, PA, USA). Reverse transcription was then performed using OneScript Hot Reverse Transcriptase (Applied Biological Materials Inc., Richmond, BC, Canada) in accordance with the manufacturer’s guidelines.

### 2.6. Quantitative Real-Time Polymerase Chain Reaction

qRT-PCR analysis was conducted using the ABI Prism 7300 system (Applied Biosystems, Thermo Fisher Scientific, Waltham, MA, USA). Amplification reactions were carried out with the SensimixPlus SYBR Master mix (Bioline, London, UK). The primers listed in Table 1 were synthesized by Bio-Fab research (Via di Castel Romano 100, Rome, Italy) and designed using Primer Express software v1.4.0 (Applied Biosystems, Thermo Fisher Scientific, Waltham, MA, USA). Gene expression levels were calculated using the 2^−ΔΔCt^ method, with normalization to the 18S rRNA housekeeping gene as the internal control.

### 2.7. ELISA

The concentrations of pro-inflammatory cytokines and MMP proteins in the supernatants of both *A. membranaceus* extract-treated and untreated HTB-94 cells were quantified using ELISA kits, following the protocols provided by the manufacturers. The Optical Density (OD) was measured at 450 nm using a microplate reader (NB-12-0035, NeBiotech, Holden, MA, USA).

### 2.8. Statistical Analysis

Results were expressed as mean ± standard error of the mean (SEM) from a minimum of at least three independent experiments, each performed in duplicate or triplicate. Statistical analysis was carried out using Prism 5.0 software (GraphPad Software, San Diego, CA, USA). A two-way repeated measures analysis of variance (ANOVA) was applied, followed by Bonferroni’s post hoc test for multiple comparisons. A *p*-value below 0.05 was considered statistically significant.

## 3. Results

### 3.1. Cytotoxicity Test

**Figure 1:**
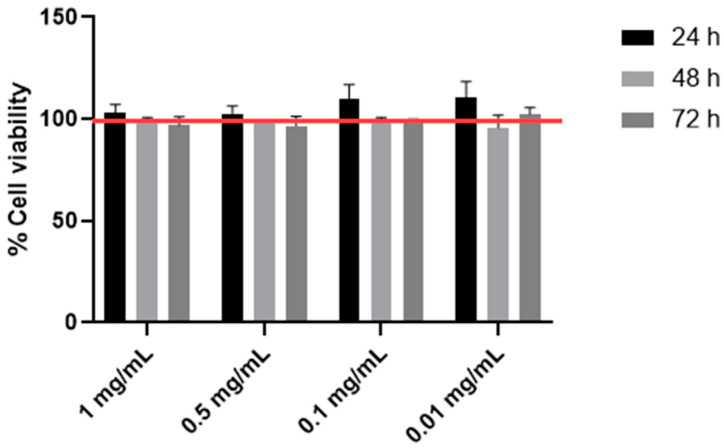
Cell viability by MTS. The viability of the HTB-94 cells treated with 0.01, 0.1, 0.5 and 1 mg/mL of *Astragalus membranaceus* extract was tested following 24, 48 and 72 h of treatment. Cell viability was normalized to that of untreated control cells, which was set at 100%, and is indicated by the horizontal red line. Data are presented as the mean ± standard deviation, based on results from three different experiments.

In order to evaluate the potential cytotoxic effect of *Astragalus membranaceus* extract, HTB-94 cells were exposed to increasing concentrations of the extract, ranging from 1 to 0.01 mg/mL, for 24, 48 and 72 h. The results obtained are shown in Figure 1. Under our experimental conditions, no cytopathic effect was observed.

### 3.2. Effects of Astragalus membranaceus Extract on the Modulation of Pro-Inflammatory Genes

**Figure 2:**
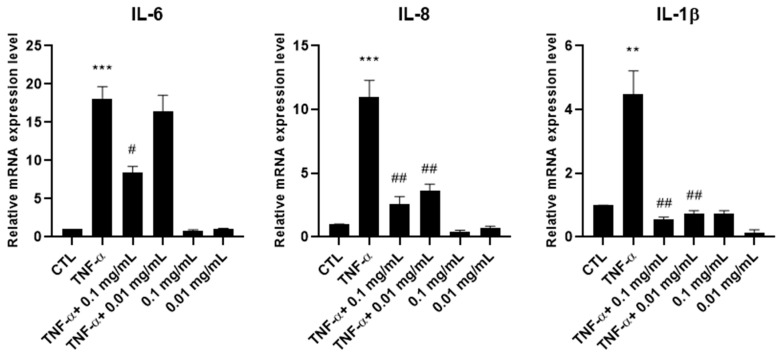
Effects of *Astragalus membranaceus* extract on interleukin mRNA expression levels. Cells were either left untreated (CTL), were exposed to 10 ng/mL TNF-α for 30 min or were pretreated with 0.01 and 0.1 mg/mL of the extract for 1 h prior to TNF-α (10 ng/mL for 30 min). Following treatment, total RNA was isolated and analyzed by RT-PCR. The mRNA expression levels of IL-6, IL-8 and IL-1β were quantified relative to 18S rRNA using the 2^−ΔΔCt^ method. Data are presented as mean ± standard deviation (SD) from three independent experiments. ** *p* < 0.01, TNF-α vs. CTL; *** *p* < 0.005, TNF-α vs. CTL; # *p* < 0.05, treated vs. TNF-α; ## *p* < 0.01, treated vs. TNF-α.

To evaluate the anti-inflammatory effectiveness of *A. membranaceus* extract, HTB-94 chondrocytes were pretreated with 0.01 and 0.1 mg/mL of the extract and subsequently stimulated with TNF-α to mimic an inflamed-joint environment. As shown in Figure 2, TNF-α stimulation increased the mRNA expression levels of IL-6, IL-8 and IL-1 β, confirming the activation of a pro-inflammatory response. Pretreatment with *A. membranaceus* extract was able to attenuate this upregulation. In particular, IL-6 expression was inhibited only at the higher concentration of *A. membranaceus* extract, while IL-8 and IL-1 β were decreased at both concentrations (0.01 and 0.1 mg/mL). Notably, treatment with *A. membranaceus* extract alone, in the absence of TNF-α stimulation, did not result in any statistically significant changes compared to the untreated control cells (CTL) (Figure 2).

### 3.3. Effects of Astragalus membranaceus Extract on Pro-Inflammatory Cytokine Secretion

**Figure 3:**
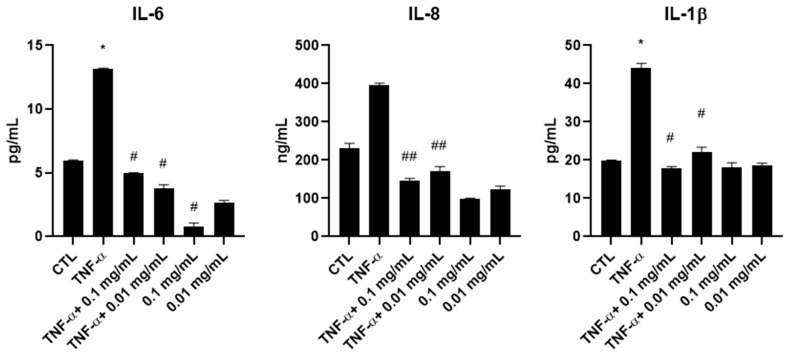
Effects of *Astragalus membranaceus* extract on interleukin secretion in the culture medium. Cells were either left untreated (CTL), were exposed to 10 ng/mL TNF-α for 1 h or were pretreated with two concentrations of extract for 1 h followed by stimulation with 10 ng/mL TNF-α for an additional hour. After the treatments, cell culture supernatants were collected and analyzed using ELISAs. Results are presented as pg/mL and expressed as mean ± SD from three independent experiments. * *p* < 0.05, TNF-α vs. CTL; # *p* < 0.05, treated vs. TNF-α; ## *p* < 0.01, treated vs. TNF-α.

To measure the effect of *A. membranaceus* extract on pro-inflammatory cytokines, ELISAs were performed on culture supernatants collected from HTB-94 cells stimulated with TNF-α and treated with 0.01 or 0.1 mg/mL *A. membranaceus* extract. Treatment with both concentrations of *A. membranaceus* extract decreased IL-6 and IL-8 production, bringing their levels below those observed in the CTL. Regarding IL-1β, treatment with 0.1 mg/mL *A. membranaceus* extract reduced the cytokine secretion to CTL levels, while 0.01 mg/mL also exerted a significant inhibitory effect, although to a slightly lesser extent (Figure 3).

Interestingly, in the absence of TNF-α stimulation, *A. membranaceus* extract at 0.1 mg/mL induced a significant reduction in IL-6 secretion below the basal CTL level. A similar trend was observed for IL-8, although the decrease was not statistically significant. In contrast, the IL-1β levels remained comparable to those of untreated cells following *A. membranaceus* extract treatment (Figure 3).

### 3.4. Effects of Astragalus membranaceus Extract on the Modulation of Metalloprotease Genes

**Figure 4:**
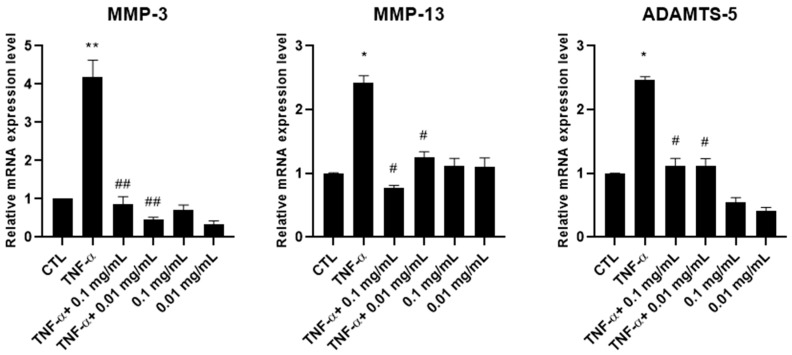
Effects of *Astragalus membranaceus* extract on MMP mRNA expression levels. Cells were either left untreated (CTL), were exposed to10 ng/mL TNF-α for 30 min or were pretreated with 0.01 and 0.1 mg/mL of the extract for 1 h prior to TNF-α stimulation (10 ng/mL for 30 min). Following treatment, total RNA was isolated and analyzed by RT-PCR. The mRNA expression levels of MMP-3, MMP-13 and ADAMTS-5 were quantified relative to 18S rRNA using the 2^−ΔΔCt^ method. Data are presented as mean ± standard deviation (SD) from three independent experiments. * *p* < 0.05, TNF-α vs. CTL; ** *p* < 0.01, TNF-α vs. CTL; # *p* < 0.05, treated vs. TNF-α; ## *p* < 0.01, treated vs. TNF-α.

We then evaluated the ability of *A. membranaceus* extract to inhibit the mRNA expression of metalloproteases (MMPs). HTB-94 cells were pretreated with 0.01 or 0.1 mg/mL of *A. membranaceus* extract, followed by stimulation with TNF-α. RNA was extracted, and the expression levels of MMP-3, MMP-13 and ADAMTS-5 were quantified by RT-PCR. The results obtained showed that, at both concentrations, the *A. membranaceus* extract significantly downregulated the mRNA expression of all the enzymes studied (Figure 4). In cells not stimulated with TNF-α, the *A. membranaceus* extract had no significant effect on the MMP-13 mRNA expression, which remained comparable to the CTL level. However, both the MMP-3 and ADAMTS-5 mRNA levels were significantly reduced below the CTL levels (Figure 4).

### 3.5. Effects of Astragalus membranaceus Extract on MMP Production

**Figure 5:**
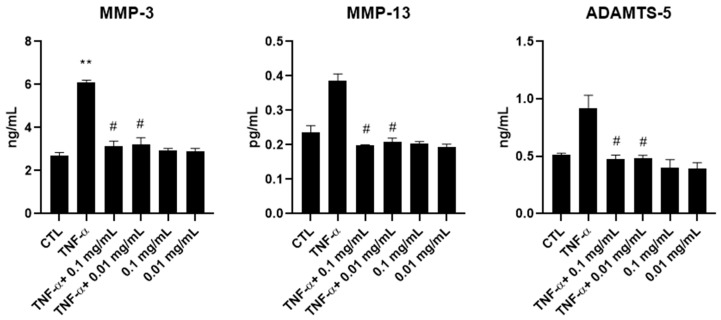
Effects of *Astragalus membranaceus* on MMP secretion in the culture medium. Cells were either left untreated (CTL), were exposed to 10 ng/mL TNF-α for 24 h or were treated with two concentrations of *A. membranaceus* extract and simultaneously stimulated with 10 ng/mL TNF-α for 24 h. Following treatment, cell culture supernatants were collected and analyzed using ELISAs. Data are presented as mean ± SD from three independent experiments. # *p* < 0.05, treated vs. TNF-α; ** *p* < 0.01, TNF-α vs. CTL.

To assess whether the reduction in MMP gene expression observed upon *A. membranaceus* extract treatment was also reflected at the protein level, the production of MMP-3, MMP-13 and ADAMTS-5 was evaluated via ELISAs. Supernatants were collected from HTB-94 cells stimulated with TNF-α and treated with 0.01 or 0.1 mg/mL of *A. membranaceus* extract. Consistent with the mRNA expression data, treatment with both concentrations of *A. membranaceus* extract effectively reduced the production of all the examined MMPs comparable to the CTL levels (Figure 5). In this case, the TNF-α-induced increase in MMP-13 and ADAMTS-5 secretion was not statistically significant. Interestingly, in cells treated with *A. membranaceus* extract without TNF-α stimulation, no significant changes in MMP secretion were observed (Figure 5).

## 4. Discussion

*Astragalus membranaceus* is a medicinal herb that is widely used in traditional Chinese medicine as an adaptogen and immunomodulator, and for the treatment of various diseases, including joint-related disorders. In recent years, its use has been investigated in the context of inflammatory and tumor diseases [38,39], with growing evidence supporting its ability to mitigate drug-induced toxicity when used in combination with conventional pharmacological treatments.

Joints are frequently affected by arthropathies, including rheumatoid arthritis, juvenile idiopathic arthritis, neuropathic arthropathy, seronegative spondyloarthropathies and OA [4,5]. These disabling pathologies are primarily driven by inflammatory processes that promote the degradation of articular cartilage, leading to pain, stiffness and the progressive loss of joint function [40].

In this study, we investigated the effects of a hydroalcoholic extract of *Astragalus membranaceus* roots on the HTB-94 cell line, demonstrating that this extract is capable of inhibiting both the gene expression and protein production of key pro-inflammatory cytokines and metalloproteases in chondrocytes under inflammatory conditions induced by TNF-α stimulation.

TNF-α was used to stimulate HTB-94 chondrocytes to mimic the inflammation typical of degenerative joint disease progression. Under inflammatory conditions, HTB-94 cells exhibited a significant increase in mRNA expression and secretion of IL-6, IL-8 and IL-1β. Treatment with *A. membranaceus* extract at 0.01 and 0.1 mg/mL significantly reduced the mRNA expression of IL-8 and IL-1β, while IL-6 was significantly decreased only at the higher concentration (0.1 mg/mL). These results suggest that *A. membranaceus* extract, by acting against inflammation of articular cartilage, may exert a protective role in joint health. These findings are of particular interest considering that the pro-inflammatory cytokines IL-6 and IL-8 are consistently found to be overexpressed in chronic joint diseases, such as RA and OA, where they play an important role in inflammation and subsequent joint damage [41]. Notably, the *A. membranaceus* extract treatment did not alter the interleukin levels in unstimulated cells, with the exception of IL-6, whose secretion was significantly reduced at 0.1 mg/mL. This finding is especially relevant, considering that IL-6 is one of the earliest mediators released in inflammatory conditions and plays a pivotal role in the activation of several pro-inflammatory pathways [42,43,44].

Articular cartilage is constituted by the extracellular matrix (ECM), consisting mainly of proteoglycans, collagen, elastin, gelatin and matrix glycoproteins [45], and contains only one cell type: chondrocytes [46]. The homeostasis of articular cartilage is tightly regulated by the balance between anabolic and catabolic processes, which is mainly mediated by matrix metalloproteases (MMPs) and their endogenous tissue inhibitors (TIMPs) [47].

Inflammatory cytokines in joints act as key mediators of MMP gene expression, stimulating both transcriptional activity and subsequent enzyme production [48].

MMPs are a class of proteolytic enzymes, classified according to the nature of their active sites and the substrates they degrade [49]. Their catalytic mechanism requires the presence of metal ions as cofactors [50]. The metalloprotease superfamily consists of different subfamilies: the MMP family, the A Disintegrin Metalloproteinase (ADAM) family and the ADAM with Thrombospondin type 1 Motif (ADAMTS) family [51]. These zinc-dependent endopeptidases are primarily responsible for the remodeling and degradation of the ECM and play a major role in the development and progression of several joint diseases [52]. While MMPs contribute to tissue homeostasis under normal physiological conditions, their expression is significantly upregulated in inflamed joints, as seen in rheumatoid arthritis and OA. In particular, MMPs may damage both collagen and proteoglycans [5,53,54], the major component of cartilage, disrupting the balance between synthesis and degradation of the ECM. The excessive enzymatic activity of MMPs and ADAMTSs leads to the progressive loss of the cartilage matrix, contributing to joint space narrowing, pain and reduced mobility, hallmarks of degenerative joint diseases [10]. Thus, strategies aimed at suppressing MMP overexpression and activity are of major interest.

In the present study, we investigated the effects of *A. membranaceus* extract on the mRNA expression levels and protein production of MMP-3, MMP-13 and ADAMTS-5 in human HTB-94 cells stimulated with TNF- α. To the best of our knowledge, this is the first manuscript studying the effects of an *A. membranaceus* extract on a chondrocyte model. Previous studies have been focused on several intracellular pathways, such as inflammatory and oxidative ones, using intestinal epithelium cells or RAW64.7 macrophages [31,55,56,57]. Moreover, *A. membranaceus* extract has also been studied as a cardioprotective and anti-diabetic agent in rat and mouse models, respectively [58,59]. To analyze the effects on the intracellular pathways involved in joint disorders, we utilized the HTB-94 cell line, which closely mimics the behavior of primary chondrocytes in terms of the MMP expression induced by inflammatory cytokines and is a well-established model for studying inflammatory pathways in chondrocytes [60]. We focused particularly on these MMPs due to their essential role in maintaining the ECM balance. MMP-3, also known as stromelysin-1, is an endopeptidase belonging to the MMP enzyme family. In normal physiological processes, MMP-3 plays a key role in the degradation of ECM proteins during tissue remodeling [61]; however, it also contributes to exacerbation of inflammation in several pathological conditions [62]. MMP-3 has been identified as a potential therapeutic target in various inflammatory diseases, including joint inflammation and OA, as well as obesity, pulmonary inflammation and periodontitis [61,63,64]. Regarding joint disease, this enzyme represents a marker of synovial inflammation and cartilage degradation, with elevated serum MMP-3 levels being closely related to the disease severity [65]. MMP-3 thus serves not only as a biomarker for monitoring disease progression but also as an indicator of therapeutic efficacy [61,66]. Like MMP-3, MMP-13 is an important mediator of cartilage degradation [54,66,67,68]. It belongs to a subgroup of MMPs known as collagenases, which are among the most effective enzymes capable of initiating the cleavage of native collagen [53]. MMP-13, also referred to as collagenase-3, is localized in articular cartilage and plays a central role in osteoarthritic joints by cleaving type II collagen, the predominant collagen in cartilage [67]. ADAMTS-5, also known as aggrecanase-2, is another key protease involved in the turnover of proteoglycans such as aggrecan and versican. Its overexpression is considered a pivotal risk factor in the progression of degenerative joint diseases [69]. Both aggrecan and versican interact with hyaluronic acid and other ECM components to form a hydrated, resilient cartilage matrix [70]. In addition, aggrecan modulates the inflammatory response through interactions with growth factors, cytokines and chemokines. Inhibiting aggrecanase activity may offer advantages over collagenase inhibition [71], as the loss of aggrecan in cartilage is one of the earliest events in the onset of joint diseases such as OA [72]. Considering the crucial role of ADAMTS-5 in the pathogeneses of joint diseases, inhibition of its expression and production could represent a promising therapeutic strategy for joint inflammatory diseases [73].

Taken together, our in vitro results demonstrate that *Astragalus membranaceus* protects cartilage by suppressing the production of pro-inflammatory cytokines and MMPs. These effects, however, are specifically attributable to the particular extract of *Astragalus membranaceus* tested in this study and cannot be generalized to other preparations without further investigation.

## 5. Conclusions

To the best of our knowledge, this is the first study demonstrating that treatment of chondrocytes with *Astragalus membranaceus* inhibits the expression and production of not only inflammatory cytokines but also of MMP-3, MMP-13 and ADAMTS-5, which play a key role in articular cartilage degradation. These findings suggest that the specific *A. membranaceus* extract used in this study may represent a promising nutraceutical approach for mitigating joint inflammation and cartilage degeneration. Our findings also suggest that this extract could be considered as a supportive strategy to maintain joint health and prevent inflammation-related damage. Further in vivo studies and clinical trials are warranted to confirm these results and explore the full potential of this extract in different populations.

## Figures and Tables

**Table 1:** List of primers used for RT-PCR. Accession numbers are indicated.

GENE	PRIMER SEQUENCES (Fw-Rv)
MMP-3 NM_002422.5	5′-CCTGGTACCCACGGAACCT-3′ 5′-AGGACAAAGCAGGATCACAGTT-3′
MMP-13 NM_002427	5′-TTCTTGTTGCTGCGCATGA-3′ 5′-TGCTCCAGGGTCCTTGGA-3′
IL-6 NM_000600	5′-GATGGATGCTTCCAATCTG-3′ 5′-CTCTAGGTATACCTCAAACTCC-3′
IL-1 β NM_000576	5′- ACGAATCTCCGACCACCACTA -3′ 5′- TCCATGGCCACAACAACTGA -3′
ADAMTS-5 NM_007038.5	5′-GCACTTCAGCCACCATCAC-3′ 5′-AGGCGAGCACAGACATCC-3′
18S NM_003286	5′-CGCCGCTAGAGGTGAAATTC-3′ 5′-CATTCTTGGCAAATGCTTTCG-3′

## Data Availability

The HTB-94 human chondrosarcoma cell line (SW1353) was obtained from the American Type Culture Collection. Data are contained within the article and are available upon request.
